# Cordycepin Enhances the Therapeutic Efficacy of Doxorubicin in Treating Triple-Negative Breast Cancer

**DOI:** 10.3390/ijms25137077

**Published:** 2024-06-27

**Authors:** Haichen Huang, Xiaomin Li, Wenya Wu, Chengyi Liu, Yunhe Shao, Xiaoping Wu, Junsheng Fu

**Affiliations:** 1 College of Life Sciences, Fujian Agriculture and Forestry University, Fuzhou 350002, China; haichenhuang312@gmail.com (H.H.); lxm2651277345@163.com (X.L.); fjwxp@126.com (X.W.); 2 Mycological Research Center, Fujian Agriculture and Forestry University, Fuzhou 350002, China

**Keywords:** triple-negative breast cancer, cordycepin, doxorubicin, network pharmacology, TNF signaling pathway

## Abstract

Triple-negative breast cancer (TNBC) is a subtype of breast cancer with high mortality and poor prognosis. Meanwhile, doxorubicin, a chemotherapeutic agent for triple-negative breast cancer, has poor sensitivity. The objective of this study was to examine the effect of cordycepin on doxorubicin sensitivity and efficacy in the TNBC xenograft model and explore the relevant molecular pathways. The combination of the drugs in nude mice carrying MDA-MB-231 xenografts significantly reduced the volume, size, and weight of xenografts and improved the tumor inhibition rate. The drug combination was significantly more effective than cordycepin or doxorubicin alone, reflecting the fact that cordycepin enhanced the anti-tumor effects of doxorubicin in MDA-MB-231 xenografts. At the same time, the monitoring of several biological parameters failed to detect any obvious side effects associated with this treatment. After predicting the importance of the TNF pathway in inhibiting tumor growth using network pharmacology methods, we verified the expression of TNF pathway targets via immunohistochemistry and quantitative PCR. Furthermore, a TNF-α inhibitor was able to abrogate the beneficial effects of cordycepin and doxorubicin treatment in MDA-MB-231 cells. This clearly indicates the role of TNF-α, or related molecules, in mediating the therapeutic benefits of the combined treatment in animals carrying TNBC xenografts. The observations reported here may present a new direction for the clinical treatment of TNBC.

## 1. Introduction

Breast cancer is the most common malignant tumor in females worldwide, ranking first among female cancers. Triple-negative breast cancer (TNBC) fails to express estrogen receptors (ERs), progesterone receptors (PRs), or the human epidermal growth factor receptor-2 (HER-2) [1]. Clinically, these tumors are characterized by recurrence and metastasis formation and are associated with high mortality and poor prognosis [2]. The mainstay of the current treatment of TNBC is chemotherapy, with doxorubicin being one of the most effective chemotherapeutic compounds [3]. However, its side effects and the emergence of drug resistance result in suboptimal outcomes. Thus, there is an urgent need to find new anti-tumor agents, preferably with low toxicity.

The use of combination drug therapies has been a major milestone in oncology and is likely to remain the leading trend in cancer treatment [4]. In addition, studies of natural drugs have identified compounds with potential anti-tumor effects and limited toxicity. There is an increasing potential for these to be used in clinical anti-tumor therapy. For example, Ginsenoside Rg3, combined with tumor statin 19 peptides, could inhibit the proliferation of HepG2 cells. This resulted in an enhanced anti-tumor effect, reduced effective drug dosage, and limited side effects [5]. Zhao et al. [6] showed that paclitaxel (PTX) combined with resveratrol (RES) can enhance the sensitivity of multi-drug resistance (MDR) cancer cell lines to paclitaxel. Thus, developing anti-tumor compounds derived from natural ingredients has the potential to enhance the effectiveness of drug combinations, providing a new direction for oncology research.

Cordycepin is an active substance isolated from *Cordyceps militaris*. It has recorded anti-tumor, antibacterial, antiviral, and immunoregulatory actions and can remove free radicals [7]. Cordycepin effectively inhibited cell proliferation and metastasis formation in lung cancer, liver cancer, prostate cancer, colon cancer, and leukemias [8]. Ma et al. [9] demonstrated that cordycepin caused apoptosis in HeLa cells by interfering with RNA synthesis. Tania et al. [10] also found that cordycepin controlled the proliferation of SiHa and HeLa cell lines by increasing apoptosis and inhibiting cell cycle progression. Thus, it appears that cordycepin has an obvious anti-tumor effect on cervical cancer cells. In addition, cordycepin can be used to potentiate the effects of other chemotherapeutic compounds while showing a remarkable safety record [11]. In experimental settings, combining cordycepin with platinum drugs (cisplatin) significantly improved the antiproliferative, migration-inhibiting, and pro-apoptotic effects of cisplatin on the SW1116 human colon cancer cell line, suggesting that cordycepin has an adjuvant effect in chemotherapy [12]. There is also evidence that the combination of cordycepin and doxorubicin shows enhanced cytotoxicity against EBV-positive tumors, while cordycepin could significantly ameliorate the deleterious effects caused by doxorubicin [13].

We have previously demonstrated that cordycepin affected the growth, apoptosis, and metastatic tendency of human breast cancer cells (MCF-7, MDA-MB-231, and MDA-MB-468 cells) and metastasis of the MDA-MB-231 xenograft in nude mice by affecting the hedgehog pathway, with low toxicity [14,15]. Furthermore, cordycepin combined with doxorubicin could inhibit the proliferation and motility of the TNBC MDA-MB-231 cell line in vitro [16]. Given the poor prognosis of TNBC and the high toxicity and poor sensitivity associated with doxorubicin monotherapy, we decided to investigate whether cordycepin enhances doxorubicin sensitivity and the effectiveness of combined cordycepin + doxorubicin therapy by establishing an MDA-MB-231 xenograft model in nude mice. In addition, we explored the mechanisms mediating the effects of the combination therapy using network pharmacology. Here, we report the mechanism of action of the combined cordycepin + doxorubicin treatment of TNBC, providing insights into the theoretical basis for the clinical treatment of TBNC. This work also lays the foundations for studying the multi-target and multi-component actions of traditional Chinese herbal compounds.

## 2. Results

### 2.1. Cordycepin + Doxorubicin Inhibited the Growth of MDA-MB-231 Xenografts in Nude Mouse Model

**Figure 1:**
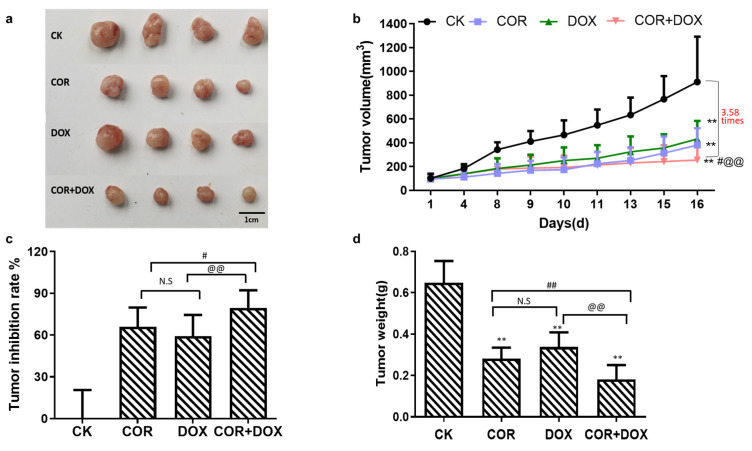
Effects of cordycepin combined with doxorubicin on a xenograft tumor. (**a**) Tumor size; (**b**) Tumor volume; (**c**) Tumor inhibition; (**d**) Tumor weight. Compared with CK group ** *p* < 0.01; compared with COR group ^#^ *p* < 0.05, ^##^ *p* < 0.01; compared with DOX group ^@@^ *p* < 0.01 and weight loss, verifying that the combination of cordycepin and doxorubicin had low toxicity in this animal model.

Xenografts of MDA-MB-231 cells were established in Balb/c nude mice, and the animals were treated with various drugs or drug combinations described in the Materials and Methods section. After 16 days, the animals were euthanized, and the xenografts were removed by dissection. All tumors were photographed, and their weight and volume were recorded. As shown in Figure 1, the smallest tumors were seen in animals treated with the combination of cordycepin + doxorubicin (COR+DOX group). Interestingly, tumors were smaller in the cordycepin (COR) group than in the doxorubicin (DOX) group (Figure 1a). Tumor growth curves were created using tumor volume data recorded during the experiment (Figure 1b). While all xenografts increased in volume over time, on day 8, the growth differences between the treatment groups and the control (CK) group were significant (*p* < 0.05). Furthermore, these differences became highly significant by day 16 (*p* < 0.01). The largest tumor volumes were seen in untreated animals. The volumes were smaller in the DOX group and even smaller in the COR group, and the smallest tumors were recovered from animals receiving the combination treatment. The difference in tumor volumes was 3.58-fold between the CK group and the animals receiving both drugs simultaneously. On day 16, compared with the CK group, the tumor volume in the administration group was significantly decreased (*p* < 0.01). Compared with the COR group, the tumor volume in the COR+DOX group was significantly reduced (*p* < 0.05). Compared with the DOX group, the tumor volume in the COR+DOX group was significantly reduced (*p* < 0.01). These results showed that the therapeutic effect of cordycepin combined with doxorubicin was better than that of cordycepin and doxorubicin alone.

When the tumor volumes were expressed as inhibition ratios, by comparing them to the control untreated MDA-MB-231 xenografts, growth was inhibited by 59.19% ± 15.30 in the DOX group, by 65.86% ± 13.88 in the COR group, and by 79.53% ± 12.63 in the COR+DOX group (Figure 1c). The COR+DOX group had the highest tumor inhibition rate, which was significantly different from that for the DOX group (*p* < 0.05) and highly significantly different from that for the COR group (*p* < 0.01).

When tumor growth was analyzed based on the weight of the dissected grafts (Figure 1d), the ascending order was the COR+DOX group < COR group < DOX group < CK group. Tumors in the control group were 3.57 times heavier than those in the combination group. There were significant differences between the three treatment groups and the CK group (*p* < 0.01) and highly significant differences between the combination and COR groups and between the COR+DOX group and the DOX group (*p* < 0.01).

The above results show that cordycepin combined with doxorubicin significantly inhibited the growth of TNBC cells. This combination was superior in its effectiveness to either of the drugs used alone, reflecting the fact that cordycepin enhances the drug sensitivity and anti-tumor efficacy of doxorubicin.

### 2.2. Cordycepin + Doxorubicin Had No Toxicity in Nude Mice

To determine whether cordycepin + doxorubicin caused obvious harm in the treated animals, we observed the vigor, body weight, and liver function of nude mice.

**Figure 2:**
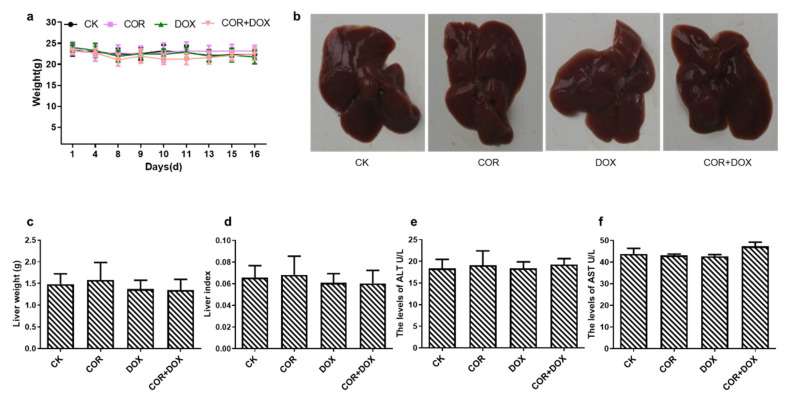
A lack of obvious toxicity of the cordycepin + doxorubicin drug combination in nude mice. (**a**) The weights of the nude mice from the different experimental groups after the completion of the treatment period. (**b**) Representative images of the hepatic morphology. (**c**) The liver weight of nude mice. (**d**) The liver index in the various treatment groups. (**e**) Serum ALT levels. (**f**) Serum AST levels.

During the experimental period, the vigor of each group of animals was observed daily, and their body weight was measured regularly. Our observations showed that the animals in each experimental group failed to exhibit any obvious abnormalities in their eating, vigor, and activity levels. The results (Figure 2a) also showed that there was no significant difference in body weight between the COR+DOX group, COR group, DOX group, and CK group (*p* > 0.05).

At the end of the experiment, the animals were dissected, and their livers were removed, observed, and photographed. The results (Figure 2b) showed that the color and texture of the livers in the three experimental treatment groups were similar to those in the control group. All the livers were rosy, uniform, and elastic.

Based on the body weight of the animals and the weight of the removed livers, the liver index was calculated in each treatment group (Figure 2c,d). These calculations showed no significant differences between the animals in the COR group, the DOX group, the COR+DOX group, and the CK group (*p* > 0.05).

We also measured serum ALT and AST levels (Figure 2e,f) and found no significant changes between the four groups (*p* > 0.05).

The above results indicate that cordycepin, doxorubicin, or the combination treatment did not cause significant liver damage and weight loss, verifying that the combination of cordycepin and doxorubicin had low toxicity in this animal model.

### 2.3. Identifying Common Molecular Targets of Cordycepin and Doxorubicin in TNBC Cells

To explore the molecular mechanism underlying the action of the drug combination, the relationships or potential targets of diseases and drugs were analyzed using network pharmacology.

**Figure 3:**
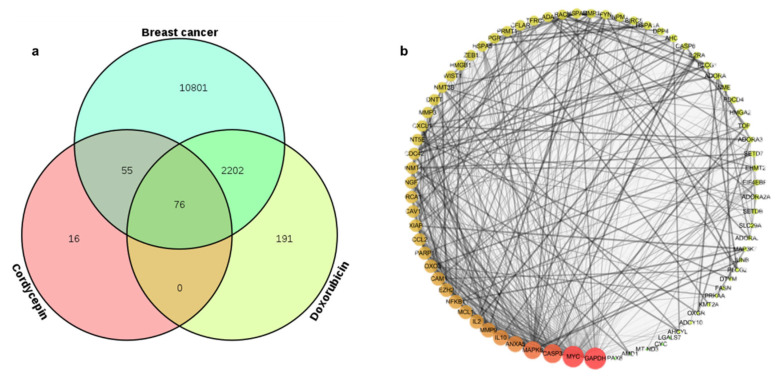
The relationship between the potential molecular targets of cordycepin and doxorubicin and breast cancer. (**a**) A Venn diagram illustrating the overlap between the potential cellular targets of cordycepin, doxorubicin, and breast cancer. (**b**) A diagram of PPI network interactions between the molecules common to the three groups.

Database searches, after the removal of duplicates, identified 147 cellular molecules that could be affected by cordycepin, 2469 molecules affected by doxorubicin, and 13,134 drug targets in triple-negative breast cancers. A Venn diagram (Figure 3a) shows 76 molecules present in all three lists, indicating the most likely potential targets (Table S1) affected by the combined cordycepin + doxorubicin treatment.

### 2.4. PPI Network Construction

After obtaining the target genes mentioned above, we needed to analyze the relationship between two or more genes. The 76 potential target genes represented by the overlapping area in the Venn diagram (Figure 3a) were analyzed against the String database to construct a PPI interaction network (Figure 3b). The resulting network consisted of 76 nodes and 532 edges. The circular nodes represent genes affected by the drugs, with lines between them indicating an interaction between the two proteins. In Figure 3b, the larger the node and the darker its color, the higher the importance of the molecule in the network. The most critical molecules affected by the two drugs correspond to the nodes representing CASP3, MAPK8, MMP9, IL2, and NFKB1. These genes appeared to mediate the most important interactions within the entire protein network. Thus, they are likely critical to the action of the cordycepin + doxorubicin drug combination in TNBC.

### 2.5. The Cordycepin + Doxorubicin Drug Combination Affects Multiple Biological Processes

**Figure 4:**
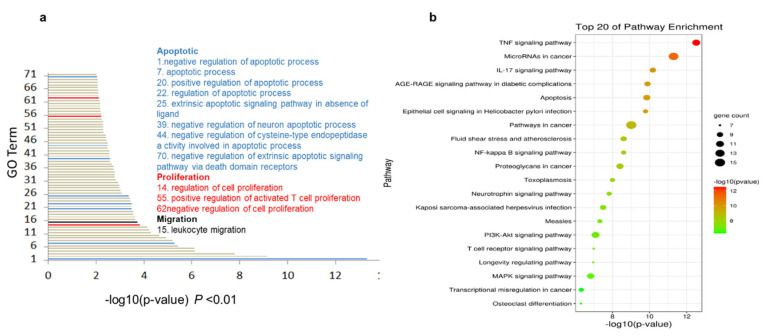
A biological function and pathway analysis of cordycepin combined with doxorubicin against breast cancer. (**a**) The biological processes of cordycepin combined with doxorubicin against breast cancer. The text on the right shows the top 20 biological processes. The blue font shows the biological process related to apoptosis; the red font indicates the biological process related to proliferation; and the black font indicates the biological process related to migration. (**b**) Top 20 pathways enriched via cordycepin combined with doxorubicin against breast cancer.

To identify the biological processes affected by the drug combination, Gene Ontology (GO) analysis was performed on the 76 intersecting proteins using the David database. The 89 GO terms with a significance of *p* < 0.01 were selected and represented on a bar graph. The results (Figure 4a) showed that the genes of the 89 biological processes were related to apoptosis (represented by the blue bar in Figure 4a). The pathways in this category included “negative regulation of apoptotic process”, “apoptotic process”, “positive regulation of apoptotic process”, and “regulation of apoptotic process”, and the next main biological process was related to cell proliferation (represented by the red bar in the graph). The pathways in this category included “negative regulation of cell proliferation”, “activated T cell proliferation”, “positive regulation of activated T cell proliferation”, and “negative regulation of cell proliferation”. In the main category of migration (represented in black), the affected process was “leucocyte migration”.

The ability of the cordycepin + doxorubicin combination to induce apoptosis, inhibit the proliferation of cancer cells, and initiate leucocyte migration into the tumor microenvironment suggests a multitude of biological processes affected by this drug combination in the treatment of TNBC.

### 2.6. KEGG Enrichment Analysis Indicates the Central Role of the TNF Pathway in Response to Cordycepin + Doxorubicin Combination Therapy

Next, we searched the KEGG database to identify the biological pathways affected by the cordycepin + doxorubicin drug combination, accepting a significance of *p* < 0.01 as the screening condition (Figure 4b). According to this search, the 67 molecules were mostly concentrated in the TNF signaling pathway, microRNA in cancer pathway, the IL-17 signaling pathway, the AGE-RAGE signaling pathway in diabetic complications, apoptosis, epithelial cell signaling in Helicobacter pylori infection, and some other tumor-related signaling pathways. Among these, the most significantly enriched pathway was TNF signaling, which is known to affect tumor migration and apoptosis [17,18].

### 2.7. Analysis of Molecular Docking between Cordycepin, Doxorubicin, and Target Proteins

The results of network pharmacology analysis indicated that the TNF signaling pathway was the most significant pathway predicted to be affected by the cordycepin + doxorubicin drug combination. To substantiate this assumption, we conducted molecular docking experiments analyzing the potential binding between cordycepin, doxorubicin, and several molecules along the TNF signaling pathway. We tested 11 targets: MAPK8, CASP3, MMP3, CCL2, CXCL1, CFLAR, MMP9, MAP3K7, JUNB, NFKB1, and ICAM1. We searched for “Breast cancer adriamycin resistance” disease targets on the GeneCards database. Most of the 11 TNF signaling pathway targets we tested were targets of breast cancer doxorubicin resistance, except for CXCL1. Studies have shown that the TNF signaling pathway may play a key role in the resistance of cordycepin combined with doxorubicin to TNBC. Therefore, molecular docking was performed using Auto Dock 4.2 software (Table 1).

**Figure 5:**
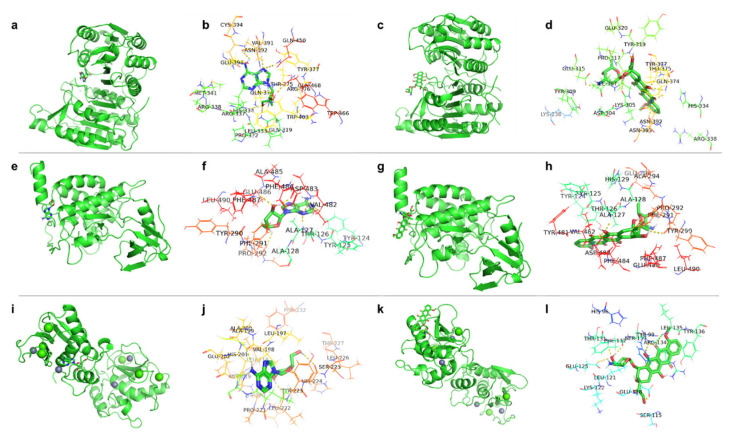
Schematic representation of molecular docking interactions. (**a**,**b**) The interactions of cordycepin with CFLAR. (**c**,**d**) The docking of doxorubicin with CFLAR. (**e**,**f**) The interactions between cordycepin and MAP3K7. (**g**,**h**) The interactions between doxorubicin and MAP3K7. (**i**,**j**) Cordycepin docking in the active pocket of MMP3. (**k**,**l**) Doxorubicin binding to MMP3.

These docking experiments identified three proteins with a binding energy of less than −5 KJ/mol and an inhibition constant of less than 1 mmol/L. The resulting interactions were visualized using PyMOL software. The results (Figure 5a–d) showed that cordycepin and doxorubicin could stably dock in the active pocket of CFLAR, an apoptosis regulator. Cordycepin formed H-bonds with amino acid residues GLN374, GLU398, and GLN468 and PI-bonds with residues GLN450, ARG337, HIS334, and GLN319, while an additional H–PI compound bond was formed with ASN392 (Figure 5a,b). On the other hand, doxorubicin established H-bonds with the GLU315 and LYS305 residues; a PI-bond with TYR319; and H–PI compound bonds with ASN392, ASN393, GLN374, and THR375 (Figure 5c,d).

Molecular docking experiments also identified stable complexes between cordycepin, doxorubicin, and the MAP3K7 protein. In the best binding conformation, cordycepin interacted with amino acid residue PRO292 via an H-bond and ALA127, TYR124, and PHE487, forming PI-bonds. In addition, H–PI bonds were formed with ASP483 and ··PHE484 residues (Figure 5e,f). In contrast, doxorubicin formed two H-bonds with PRO292 and TYR290, and PI-bonds were formed with ASP483 and ALA127 (Figure 5g,h).

The third interacting protein was MMP3. In its best complex conformation, cordycepin interacted with MMP3 via the VAL198, GLU202, and LEU22 residues through H-bonds and with HIS224 through an H–PI composite bond (Figure 5i,j). Doxorubicin formed two hydrogen bonds with MMP3 via GLU125 and GLU118, a PI-bond with ARG134, and an H–PI compound bond with PHE132 (Figure 5k,l).

The complexes described above showed that cordycepin and doxorubicin could interact with protein targets through different residues within the active site. The formation of these stable complexes suggests that the two molecules do not compete with each other for binding but might act synergistically in inhibiting the growth of TNBC cells.

### 2.8. Cordycepin + Doxorubicin Affect the Expression of Molecules in the TNF Signaling Pathway

**Figure 6:**
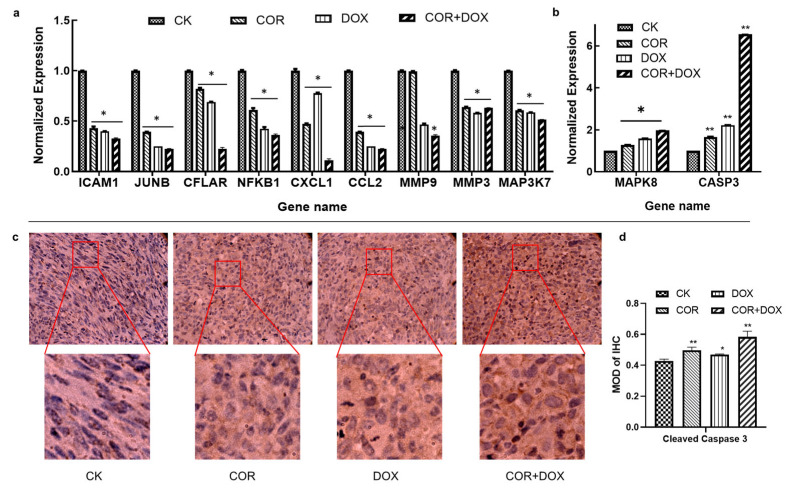
An analysis of the expression of TNF signaling pathway molecules in breast cancer. (**a**,**b**) The abundance of mRNAs studied using qPCR. * *p* < 0.05, ** *p* < 0.01. (**c**) The expression of Cleaved Caspase 3 detected via immunohistochemistry. The magnification factor is 10 × 20. (**d**) Image J software analyzed the mean optical density values of Cleaved Caspase 3 immunohistochemistry.

Since the molecular docking models confirmed the interaction of three proteins of the TNF pathway with the cordycepin + doxorubicin drug combination, we next examined the effect of this drug combination on the expression levels of molecules involved in this pathway using quantitative PCR. These experiments indicated that, compared with the control group, the drug treatment resulted in significant downregulation in the abundance of ICAM1, JUNB, CXCL1, CCL2, MMP3, CFLAR, NFKB1, MMP9, and MAP3K7, resulting in significant upregulation in the abundance of MAPK8 and CASP3, and the regulating effect of the combined group was the most significant (Figure 6a,b). The most profound effect of cordycepin treatment was seen in JUNB expression, resulting in a 2.54-fold reduction in abundance. The most pronounced effect of doxorubicin was the 4.01-fold downregulation in CCL2 expression. Compared with the control group, the combination of cordycepin + doxorubicin had the most significant effect on upregulating the expression of the CASP3 gene (*p* < 0.01), which was 3.94-fold and 2.99-fold that of the COR group and the DOX group, respectively.

The upregulation in Cleaved Caspase 3 was also confirmed at the protein level using immunohistochemistry. Staining with a Cleaved Caspase 3 antibody detected a significant increase in Cleaved Caspase 3 signal in cordycepin + doxorubicin-treated MDA-MB-231 xenograft tissues (Figure 6c). Compared with the CK group, there were significant differences in the positive rates of Cleaved Caspase 3 protein between the cordycepin group and the COR+DOX group (*p* < 0.01), and there were significant differences in the DOX group (Figure 6d).

These qPCR and immunohistochemistry data verified our network-pharmacology-based conclusions, indicating that cordycepin and doxorubicin modulate multiple target genes in the TNF signaling pathway in vivo in TNBC cells.

### 2.9. The TNF-α Inhibitor Pomalidomide Reverses the Inhibitory Effect of Combined Cordycepin + Doxorubicin Treatment on MDA-MB-231 Cells

**Figure 7:**
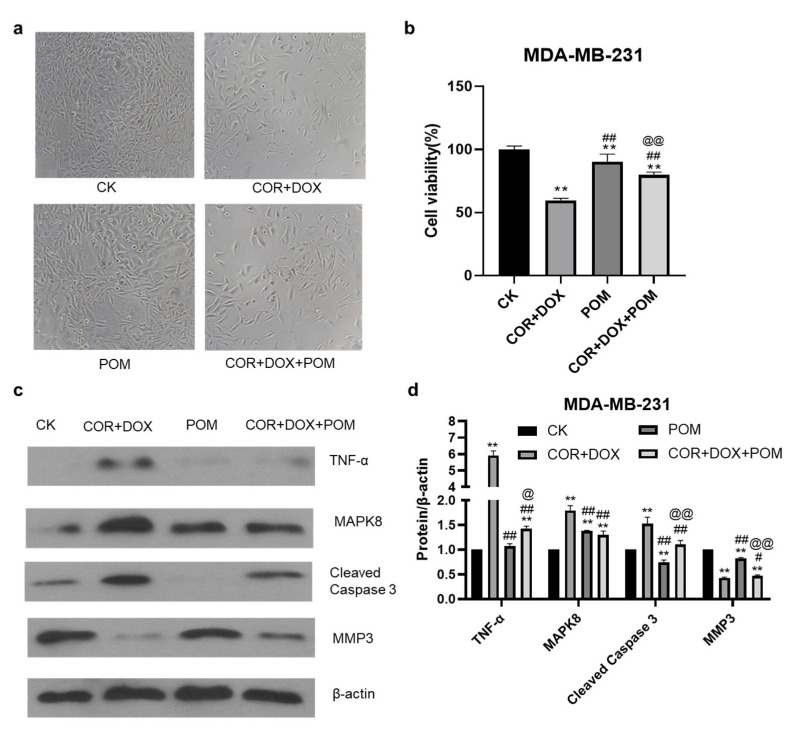
The effect of a TNF inhibitor inhibits the TNF pathway in MDA-MB-231 cells. (**a**) The morphology and quantity of MDA-MB-231 cells. (**b**) Cell viability. (**c**) Western blot analysis of proteins in the TNF pathway. (**d**) The expression of TNF-α, MAPK8, Cleaved Caspase 3, and MMP3 proteins. **: compared to control (CK) group, *p* < 0.01, ##: compared to the COR+DOX group, # *p* < 0.05, *p* < 0.01, @: compared to the POM group, *p* < 0.05, @@: compared to the POM group, *p* < 0.01.

To demonstrate the biological relevance of the TNF pathway in the combined cordycepin + doxorubicin treatment of TNBC, we tested whether their effects were inhibited in the presence of a TNF-α inhibitor, pomalidomide. In these experiments, MDA-MB-231 cells were cultured in vitro, and the effect of drug treatment on their viability was assessed using the MTT cytotoxicity assay. The results showed that the inhibitory effect of cordycepin + doxorubicin profoundly reduced the survival of MDA-MB-231 cells in culture. However, the addition of the TNF inhibitor, pomalidomide, reversed the inhibitory effects of the drug combination, increasing the viability of MDA-MB-231 cells (Figure 7a,b). Thus, it appears that an intact TNF-α signaling pathway is essential to mediate the inhibitory effects of the combined drug treatment. The above results indicate that the TNF pathway plays an important regulatory role in inhibiting breast cancer growth caused by cordycepin combined with doxorubicin.

### 2.10. The Inhibition of the TNF-α Pathway Inhibitor Eliminates Changes in Protein Abundance Induced by the Combination Treatment of MDA-MB-231 Cells

To further investigate the role of the TNF signaling pathway in the treatment of TNBC, we examined the effects of the TNF-α inhibitor, pomalidomide, on Cleaved Caspase 3, TNF-α, MAPK8, and MMP3 protein expression using Western blot analysis (Figure 7c). In these experiments, a clear upregulation in the expression of TNF-α, MAPK8, and Cleaved Caspase 3 was seen after combined treatment with cordycepin + doxorubicin, and the treatment inhibited the expression of MMP3, in agreement with previous qPCR experiments. However, when the TNF-α inhibitor was added to MDA-MB-231 cultures treated with the drug combination, the upregulation in TNF-α, MAPK8, and Cleaved Caspase 3 and the inhibition of MMP3 were almost completely abrogated. The ability of a TNF-α inhibitor to almost completely reverse the effect of the combined drug treatment on protein expression by MDA-MB-231 lends additional support to the importance of the TNF signaling pathway in mediating the effects of cordycepin + doxorubicin treatment against TNBC.

### 2.11. Analysis of the Target Pathway Network

**Figure 8:**
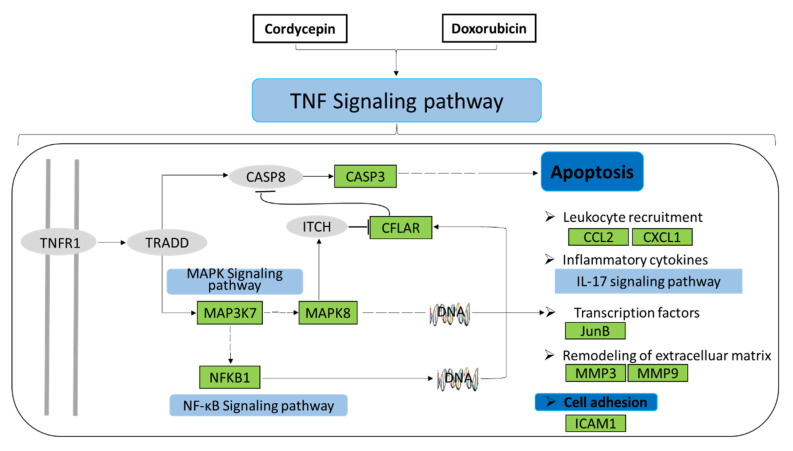
Potential target pathway analysis of cordycepin combined with doxorubicin against breast cancer. Green indicates the predicted effective target; blue indicates the predicted effective biological process; light blue indicates the predicted effective signal pathway; and gray indicates the potential target to be confirmed.

Combining the KEGG pathway enrichment data, the results of molecular docking models, our experimental observations, and data from the literature allowed us to draw the final pathway map summarizing the action of the combined cordycepin + doxorubicin treatment in TNBC. As illustrated in Figure 8, the combination of cordycepin and doxorubicin regulates the MAPK and NF-κB signaling pathways, IL-17 signaling, and apoptosis. Critically, TNF signaling via MAP3K7, NFKB1, MAPK8, CFLAR, CASP3, CCL2, CXCL1, JUNB, MMP3, MMP9, and ICAM1 caused apoptosis and inhibited metastasis formation by MBA-MD-231 cells. These findings may support a central role for TNF-mediated mechanisms being responsible for the beneficial effects of the combined treatment in the management of TNBC in our xenograft model. These observations may open new avenues in the clinical use of these compounds.

## 3. Discussion

Cordycepin is a traditional Chinese medication. It has been reported to exhibit antibacterial, anti-inflammatory, anti-tumor, and antiviral effects while having a good safety record [11]. Its anti-tumor effects are supported by several experimental studies. For example, cordycepin can affect the proliferation, migration, invasion, cell cycle, and apoptosis of lung cancer. It was used to solve the problems associated with gefitinib and anti-resistance in the treatment of lung cancer [19]. It was also used to help develop radiation resistance in some cancer cells [20]. Currently, chemotherapy is the main treatment option in the treatment of TNBC. However, the current clinically used chemotherapeutic compounds have several disadvantages, including the emergence of drug resistance, high toxicity, and adverse reactions. Cordycepin is the main active component of *Cordyceps militaris*. There have been no relevant reports on the acute and chronic toxicity of cordycepin so far [21]. Doxorubicin is a kind of chemotherapy drug that has adverse side effects on the human body; therefore, it has become a new research trend to reduce the dose and use it in combination with other anticancer drugs. Du et al. [13] discovered that cordycepin can obviously enhance the EBV-positive tumor treatment efficacy via low-dose doxorubicin. Combination therapy, with the simultaneous or sequential use of drugs, can overcome some of these limitations. As a natural product, cordycepin is a good potential choice for inclusion in such combined drug therapy due to its effectiveness, safety, and low toxicity. For example, the combination of cordycepin and gemcitabine was used successfully to significantly inhibit the growth of breast cancer cells and promote apoptosis [22]. Breast cancer is a major cause of death for women and seriously affects women’s health. Triple-negative breast cancer is the subtype with the worst prognosis among breast cancers. It is poorly differentiated, easily metastasizes, and lacks targets. Our preliminary studies have found that cordycepin can significantly inhibit the growth of MDA-MB-231 xenograft tumors [15]. Therefore, this study examined in depth the effect of combined drugs on triple-negative breast cancer. These considerations led us to test cordycepin + doxorubicin’s combination in a mouse model of TNBC xenografts. The results showed that this drug combination did not affect the growth, weight, or liver function of nude mice, suggesting good short-term safety and low toxicity. Furthermore, cordycepin + doxorubicin’s combination was significantly more effective than either compound alone in reducing both the weight and size of the developing MDA-MB-231 xenograft tumors, resulting in an improved tumor inhibition rate. Network pharmacology is a relatively new approach facilitating studies into the complex actions of traditional Chinese formulations. Klee et al. [23] studied the mechanism of action of traditional drugs in epilepsy utilizing network pharmacology. Given the complex etiology of this condition and the limitations of single-drug therapy, they proposed a strategy to evaluate the rational selection of drug combinations for the management of epilepsy. Using network pharmacology, Liu et al. [24] discovered the signaling pathways that XYP acts upon in anovulatory infertility. This approach identified novel therapeutic targets in this condition. Utilizing a similar approach, we defined the molecular pathways that the combination of cordycepin + doxorubicin is likely to affect in TNBC. GO and KEGG enrichment analyses of these pathways indicated that the TNF signaling pathway, microRNAs, IL-17 signaling, the AGE-RAGE signaling pathway, and apoptosis-related pathways were the most critically relevant. Of these, the TNF pathway was the most enriched, affecting 11 potential protein targets, including MAPK8, CASP3, MMP3, CCL2, CXCL1, CFLAR, MMP9, MAP3K7, JUNB, NFKB1, and ICAM1.

Data from the literature indicate that TNF-α can trigger the activation of pathways related to the inhibition of tumor growth, including signaling via the NF-κB and MAPK pathways [25]. NF-κB plays an important role in regulating cellular proliferation, differentiation, and invasive and metastatic behavior in tumor cells. The activation of NF-κB can be observed in various malignant human tumors. Regarding the other molecular pathways identified in our work, the abnormal expression of miRNAs is related to the biological behavior of tumor cells and was implicated in cell proliferation, invasive and metastatic behavior, angiogenesis, drug resistance, and the modification of signaling pathways. IL-17 participates in anti-tumor immune responses via the regulation of T-cell immunity [26,27,28,29]. Studies have shown that cordycepin could inhibit TNF-signaling-induced MAPK activation, the expression of CFLAR, and regulate the CASP family and MMP family of proteins, thereby inhibiting tumor proliferation and metastasis while promoting apoptosis and autophagy in TNBC cells [30,31,32]. After predicting the central role of the TNF pathway using network pharmacology methods in the treatment of TNBC via the drug combination of cordycepin + doxorubicin, 11 proteins of the TNF pathway were studied for their ability to interact with these two drugs. Using molecular docking, we could identify three proteins of the pathway, CFCLAR, MAP3K7, and MMP3 cordycepin, that doxorubicin could interact with. Additional qPCR and immunohistochemistry experiments found that cordycepin, doxorubicin, or a combination of the two drugs had a significant effect on the expression of other molecules of the TNF signaling pathway. Of these, the most striking effect was the upregulation in Cleaved Caspase 3. Finally, using a TNF-α inhibitor, we were able to confirm that the inhibitory effects of the drug combination on MDA-MB-231 cells were primarily mediated via the TNF pathway.

The work presented here confirms that cordycepin combined with doxorubicin has a remarkable anti-tumor effect against a TNBC-derived cell line and TNBC xenografts. At the same time, during the experimental period, we failed to detect any evidence of obvious toxicity. Utilizing network pharmacology, we predicted that genes involved in the TNF pathway played a central role in mediating the beneficial effects of the drug combination. By measuring the expression of related target molecules and exploring the effect of a TNF-α inhibitor, we were able to experimentally verify the central role of the TNF pathway in the anti-tumor activity of the cordycepin and doxorubicin drug combination.

## 4. Materials and Methods

### 4.1. Cells, Animals, and Chemical Compounds

The human triple-negative breast cancer cell line, MDA-MB-231, was purchased from iCell Bioscience Inc. Cordycepin was purchased from Sigma (San Francisco, CA, USA). Forty female SPF-grade 3–5-week-old Balb/c nude mice weighing 14–15 g were obtained from Wu’s laboratory animal Online (http://www.wssydw.com/m/index.asp, accessed on 23 June 2024). RPMI 1640 medium, fetal bovine serum, green/streptomycin, and Matrigel matrix were purchased from Gibco (Grand Island, NY, USA); Trypsin was provided by Beijing Solarbio Science & Technology, Ltd.; and 4% paraformaldehyde, sodium chloride, potassium chloride, potassium dihydrogen phosphate, phenol, isopropanol, and 30% hydrogen peroxide were supplied by Sinopharm Chemical Reagent, Ltd. The ALT and AST detection kits were purchased from Nanjing Jiancheng Bioengineering Institute, and the Trizol RNA Extraction Kit was purchased from Invitrogen (Carlsbad, CA, USA). The SYBR green qPCR reverse transcription kit was purchased from TransGen Biotech; the citrate buffer was obtained from Servicebio. The hematoxylin staining solution, neutral gum, the HRP labeled goat anti-rabbit secondary antibody, the TNF-α inhibitor Pomalidomide, and the DAB chromogenic histochemistry reagents were all obtained from the Beyotime Institute of Biotechnology. The Cleaved Caspase 3, TNF-α, MAPK8, MMP3, and β-actin primary antibodies were purchased from Proteintech Group. The ready-to-use SABC-AP kit was purchased from BOSTER Biological Technology Co., Ltd.

### 4.2. Cell Culture

The MDA-MB-231 human triple-negative breast cancer cells were cultivated in RPMI 1640 medium containing 10% fetal bovine serum and 1% Penicillin–Streptomycin Liquid. Flasks were incubated in 5% CO_2_ at 37 °C.

### 4.3. Xenograft Tumor Model

MDA-MB-231 cells in the logarithmic growth phase were digested with trypsin, diluted to a cell suspension of 1 × 10^8^ cells/mL, and injected subcutaneously into nude mice. Each nude mouse was injected with 200 μL of cell suspension. Based on our previous in vitro experiments and data from the literature, doxorubicin was administered at 5 mg/kg and cordycepin at 20 mg/kg. When the tumors reached approximately 100 mm^3^, the animals were randomized into 4 groups, with 10 nude mice in each group. The control group (CK) received sterile water (ig) every day and an intraperitoneal injection of normal saline once a week; the cordycepin (COR) group was given 20 mg/kg of the drug (ig) every day; the doxorubicin (DOX) single-compound group was administered a dose of 5 mg/kg (ip) once a week; the combination group was administered cordycepin (ig) at 20 mg/kg per day, and 5 mg/kg of doxorubicin was injected (ip) once a week, with continuous administration for 16 days. After the last dose, animals were fasted but were allowed to drink. After 24 h, blood was taken from the eyeball and centrifuged to obtain a serum. The animals were euthanized; the tumors and livers were removed; and various physiological indicators were recorded.

### 4.4. Analysis of Hepatotoxicity

All experimental animals were weighed daily, and their behaviors, such as eating and general activity, were recorded. At the end of the experimental period, mice were dissected, and the livers were removed and rinsed in normal saline. After draining the excess fluid, livers were observed for color, weighed, and the liver index was calculated. Serum ALT and AST were measured according to the manufacturer’s instructions and calculated against a standard curve for each enzyme.

### 4.5. Prediction of Relevant Targets

Referring to and organizing the public databases used by Zhang et al. and Wen et al., this study combined multiple databases to search for potential targets [33,34]. Using database searches, a list of potential cellular interacting partners of cordycepin and doxorubicin was compiled. The PubChem (http://pubchem.ncbi.nlm.nih.gov, accessed on 23 June 2024), SEA (http://sea.bkslab.org/, accessed on 23 June 2024), Superpred (http://prediction.charite.de/index.php/, accessed on 23 June 2024), and GeneCards databases (https://www.genecards.org/, accessed on 23 June 2024) were used in these searches. Potentially inconsistent designations of the identified molecules were checked against the Uniprot database (https://www.uniprot.org/, accessed on 23 June 2024) for standardized protein and gene names. Breast-cancer-related target genes were identified from the GeneCards database (https://www.genecards.org/, accessed on 23 June 2024). Using the “SUMIF” function in Excel, the three lists were intersected. The molecules present in all three lists were identified. The relationship between the constituents of the three lists was illustrated by drawing a Venn diagram using OmicShare (https://www.omicshare.com/tools/, accessed on 23 June 2024).

### 4.6. Protein Interaction Analysis

We imported the three lists described in Section 4.5 into the String online database (https://string-db.org/, accessed on 23 June 2024) and set “Homo sapiens” as the species to search for interactions between them. The Network Analyzer tool in Cytoscape 3.6.0 was used to construct and analyze the network and classify it according to degree and betweenness centrality. The PPI protein interaction network diagram was drawn using Cytoscape 3.6.0.

### 4.7. GO Analysis and KEGG Analysis

The GO and KEGG classifications of 76 intersecting targets were analyzed in the David database, with *p* < 0.01 accepted as the screening condition for statistical difference. The GO term classification of the identified genes was represented in a bar graph, where the blue column represented biological processes related to apoptosis; red indicated the pathways in cell proliferation; and black marked the pathways involved in migration. KEGG pathway enrichment analysis also used *p* < 0.01 as the screening criterion for statistical difference. The top 20 identified pathways were presented as a bubble chart.

### 4.8. Molecular Docking

To identify the proteins potentially binding cordycepin and doxorubicin and to study the affinity of these interactions, we used a network pharmacology approach. Here, cordycepin and doxorubicin were treated as “ligands”, while proteins from the most heavily enriched KEGG pathways were used as potential receptors. First, the Molegro Virtual Docker 5.5 tool was used to optimize the structure of the presumed ligand and receptor pairs. Then, Auto Dock 4.2 software was utilized to carry out molecular docking experiments in silico. We used the Auto Grid function in Auto Dock 4.2 to calculate the binding energy (BE), inhibition constant (IC), and other calculated energy values. PyMOL 2.3 was used to create graphic representations of the most probable docking conformations.

### 4.9. Quantitative PCR

A trizol reagent was added to the dissected, ground-up tumor tissues, and RNA was extracted according to the manufacturer’s instructions. To synthesize first-strand cDNA, the RNA samples were diluted to 500 ng/µL and reverse-transcribed, as described in the TransScript All-in-one First-Strand cDNA Synthesis SuperMix I instructions. Primer5 software was used to design the primers, which were synthesized by Shanghai Sangon Biotech Co. Ltd. The cDNA samples were diluted 5-fold, following the instructions in the TransStart Top Green qRCR SuperMix kit. The PCR reaction conditions included denaturation (94 °C, 5 s), annealing (58 °C, 30 s), and extension (60 °C, 5 s). A total of 40 amplification cycles were performed. The results were analyzed according to the 2^−△△CT^ method to obtain quantitative results. Each experiment was performed in triplicate biological repeats.

### 4.10. Immunohistochemistry

Tumor tissue portions from each group were fixed with 4% paraformaldehyde for subsequent IHC testing. Sections from each group were deparaffinized, rehydrated, quenched with endogenous peroxidase, underwent antigen retrieval, and were blocked using xylene, graded ethanol, H_2_O_2_, citrate buffer, and 10% Goat Serum. After the Cleaved Caspase 3 antibody was added, the samples were incubated overnight in a humid chamber at 4 °C, rinsed twice with PBS, and the HRP-labeled goat anti-rabbit secondary antibody was added. Slides were incubated at 37 °C for 30 min. A drop of Streptavidin-alkaline phosphatase (SABC) was added at this point, and an additional incubation was carried out at 37 °C for 30 min. The SABC was removed via aspiration; the DAB solution was added; and the staining was terminated when red-brown substances appeared. Slides were rinsed twice with PBS, stained with hematoxylin for 10 min, treated with 1% hydrochloric acid in alcohol for a few seconds, and placed in 1% ammonia in water for 10 min. Slides were observed, and a digital camera was used to take microphotographs. Each experiment was performed in triplicate biological repeats.

### 4.11. Assessing Cellular Toxicity Using MTT Assays

We referred to the method of Plumb [35] and modified it slightly. MDA-MB-231 cells in the logarithmic growth phase were diluted into 96-well plates with 5 × 10^4^ cells/well. After the cells adhered to the wall, the drugs or drug combinations were selected as follows: 80 μM of cordycepin + 1 uM doxorubicin was added to the COR+DOX wells; 3 nM of pomalidomide (POM) was added to the POM wells; and 80 μM c of ordycepin+1 μM doxorubicin+3 nM of pomalidomide was added to the COR+DOX+POM wells. A drug-free control group was also set up, and cell-free zero-adjustment wells were also reserved. Each treatment group consisted of six parallel wells. Plates were incubated at 37 °C in 5% CO_2_ for 48 h. After this incubation, 20 μL of MTT (5 mg/mL) was added to each well and then incubated for 4 h. Then, the culture medium was discarded; 150 μL of DMSO was added; and the plates were shaken until the purple-brown precipitate completely dissolved. The OD value of each well was measured at a wavelength of 490 nm, and the cellular survival rates were calculated. Each experiment was performed in triplicate biological repeats.

### 4.12. Western Blot Analysis

BSA and Coomassie brilliant blue were used to prepare a protein standard curve. Cell lysates were diluted to the desired concentration and separated on a 10% polyacrylamide gel. After electrophoresis, proteins were transferred to PVDA membranes that were washed three times in TBST. After the addition of appropriately diluted primary antibodies (Cleaved Caspase 3, TNF-α, MAPK8, MMP3, and β-actin), the membranes were incubated overnight at 4 °C. The next day, membranes were washed three times in TBST and incubated with the HRP conjugated goat anti-rabbit IgG (H+L) for 2 h. Color development was performed in the dark for 10 min using the BCIP/NBT color development kit. Membranes were photographed using a gel imaging system to record the results. Each experiment was performed in triplicate biological repeats.

### 4.13. Construction of the Compound–Pathway–Target Network

Combining experimental findings with data from the literature, we analyzed the most likely pathways in breast cancer treatment and identified the potential targets affected by the cordycepin + doxorubicin drug combination. The resulting diagram shows the interactions between cordycepin, doxorubicin, the molecular pathways affected by the compounds, and the individual protein targets within those pathways.

### 4.14. Statistical Analysis

All data were processed using the *SPSS 25.0* statistical software package, and the results were expressed as “mean ± standard deviation (x ± s)”, using a *t*-test, with *p* < 0.05 accepted as a significant difference between the groups.

## 5. Conclusions

The results confirmed that cordycepin enhances doxorubicin sensitivity and its anti-tumor effect on TNBC xenografts without any apparent toxicity. We predicted and validated that the TNF pathway played a central role in mediating the beneficial effects of cordycepin + doxorubicin. These findings provide theoretical and experimental support for the development and clinical utilization of cordycepin as a novel therapeutic approach in the management of TNBC.

## Figures and Tables

**Table 1:** Docking of cordycepin and doxorubicin with eleven targets in the TNF signaling pathway.

Target	Binding Energy (KJ/mol)		Inhib Constant (μmol/L)	
	Cordycepin	Doxorubicin	Cordycepin	Doxorubicin
CFLAR	−7.05	−6.88	6.77	9.05
NFKB1	−5.22	−5.56	1.48 × 10^2^	84.44
CASP3	−5.21	−7.7	1.58 × 10^2^	2.28
MAP3K7	−5.96	−6.92	42.84	8.44
ICAM1	−5.22	−5.86	1.44 × 10^3^	50.42
JUNB	−3.45	−2.14	2.98 × 10^3^	2.71 × 10^4^
MMP9	−4.25	−6.48	7.62 × 10^2^	17.93
CXCL1	−4.34	−5.13	6.57 × 10^2^	1.73 × 10^2^
MAPK8	−5.37	−5.67	1.16 × 10^2^	69.51
MMP3	−6.75	−8.92	11.24	0.29
CCL2	−4.43	−4.84	5.63 × 10^2^	2.82 × 10^2^

## Data Availability

The original contributions presented in the study are included in the article/Supplementary Materials, further inquiries can be directed to the corresponding author/s.

## Supplementary Materials

The following supporting information can be downloaded at: https://www.mdpi.com/article/10.3390/ijms25137077/s1.
