# Supplementary material for: Cordycepin Enhances the Therapeutic Efficacy of Doxorubicin in Treating Triple-Negative Breast Cancer
Source: Int J Mol Sci. 2024 Jun 27;25(13):7077. doi: 10.3390/ijms25137077 (PMC11241178; doi:10.3390/ijms25137077)
Supplement: Supplementary file 1 [file ijms-25-07077-s001.zip › Table S1.pdf]

Supplementary material

Table S1. Candidate targets of Cordycepin combined with Doxorubicin against breast cancer

Uniprot ID	Target Gene	Target Protein
P00813	ADA	Adenosine deaminase
P04053	TDT	DNA nucleotidylexotransferase
P09874	PARP1	Poly ADP-ribose polymerase 1
P03956	MMP1	Interstitial collagenase
P08254	MMP3	Stromelysin-1
P19838	NFKB1	Nuclear factor NF-kappa-B p105 subunit
P16885	PLCG2	1-phosphatidylinositol 4,5-bisphosphate phosphodiesterase gamma-2
O15519	CFLAR	CASP8 and FADD-like apoptosis regulator
P38398	BRCA1	Breast cancer type 1 susceptibility protein
P37275	ZEB1	Zinc finger E-box-binding homeobox 1
P22301	IL10	Interleukin-10
P60568	IL2	Interleukin-2
Q15672	TWIST1	Twist-related protein 1
P52926	HMGA2	High mobility group protein HMGI-C
P01589	IL2RA	Interleukin-2 receptor subunit alpha
P05362	ICAM1	Intercellular adhesion molecule 1
P02786	TFRC	Transferrin receptor protein 1
Q13131	PRKAA1	5'-AMP-activated protein kinase catalytic subunit alpha-1
P14780	MMP9	Matrix metalloproteinase-9
P13500	CCL2	C-C motif chemokine 2
P09341	CXCL1	Growth-regulated alpha protein
P0DMS8	ADORA3	Adenosine receptor A3
P09429	HMGB1	High mobility group protein B1
P01138	NGF	Beta-nerve growth factor
P11387	TOP1	DNA topoisomerase 1
P01106	MYC	Myc proto-oncogene protein
P45983	MAPK8	Mitogen-activated protein kinase 8
Q07820	MCL1	Induced myeloid leukemia cell differentiation protein Mcl-1
Q03135	CAV1	Caveolin-1
P98170	XIAP	E3 ubiquitin-protein ligase XIAP
O15392	BIRC5	Baculoviral IAP repeat-containing protein 5
O43524	FOXO3	Forkhead box protein O3
P55212	CASP6	Caspase-6
P42574	CASP3	Caspase-3
P08758	ANXA5	Annexin A5
P0DMV8	HSPA1A	Heat shock 70 kDa protein 1A
P06401	PGR NR3C3	Progesterone receptor
P49327	FASN	Fatty acid synthase
P06748	NPM1	Nucleophosmin
P06241	FYN	Tyrosine-protein kinase Fyn
Q12879	GRIN2A	Glutamate receptor ionotropic, NMDA 2A
Q13541	EIF4EBP1	Eukaryotic translation initiation factor 4E-binding protein 1
P08574	CYC1	Cytochrome c1, heme protein, mitochondrial
P17275	JUNB	Transcription factor jun-B
P47929	LGALS7	Galectin-7
P03897	MT-ND3	NADH-ubiquinone oxidoreductase chain 3
P23526	AHCY	Adenosylhomocysteinase
Q96P68	OXGR1	2-oxoglutarate receptor 1

P11142	HSPA8	Heat shock cognate 71 kDa protein
P30542	ADORA1	Adenosine receptor A1
P29274	ADORA2A	Adenosine receptor A2a
Q99873	PRMT1	Protein arginine N-methyltransferase 1
P26358	DNMT1	DNA (cytosine-5)-methyltransferase 1
Q9UBC3	DNMT3B	DNA (cytosine-5)-methyltransferase 3B
P04406	Gapdh	Glyceraldehyde-3-phosphate dehydrogenase
P23109	AMPD1	AMP deaminase 1
P21589	NT5E	5'-nucleotidase
P29275	ADORA2B	Adenosine receptor A2b
Q96PN6	ADCY10	Adenylate cyclase type 10
P11021	HSPA5	Endoplasmic reticulum chaperone BiP
P60953	CDC42	Cell division control protein 42 homolog
Q96KQ7	EHMT2	Histone-lysine N-methyltransferase EHMT2
Q15910	EZH2	Histone-lysine N-methyltransferase EZH2
Q03164	KMT2A	Histone-lysine N-methyltransferase 2A
P23919	DTYMK	Thymidylate kinase
O43318	MAP3K7	Mitogen-activated protein kinase kinase kinase 7
Q06710	PAX8	Paired box protein Pax-8
Q53EL6	PDCD4	Programmed cell death protein 4
P19174	PLCG1	1-phosphatidylinositol 4,5-bisphosphate phosphodiesterase gamma-1
P63000	RAC1	Ras-related C3 botulinum toxin substrate 1
Q99808	SLC29A1	Equilibrative nucleoside transporter 1
O43865	AHCYL1	S-adenosylhomocysteine hydrolase-like protein 1
Q15047	SETDB1	Histone-lysine N-methyltransferase SETDB1
Q8WTS6	SETD7	Histone-lysine N-methyltransferase SETD7
O94759	TRPM2	Transient receptor potential cation channel subfamily M member 2
P27487	DPP4	Dipeptidyl peptidase 4
